# Psychosocial Challenges and Health-Related Quality of Life in Elderly Cancer Patients Receiving Chemotherapy: Insights From a Prospective Cohort Study

**DOI:** 10.7759/cureus.105770

**Published:** 2026-03-24

**Authors:** Priyanka Srivastava, Barna Ganguly, Ajay G Phatak, Nirav N Asarawala, Bharat M Gajjar

**Affiliations:** 1 Medical Oncology, Rayos Comprehensive Cancer Care, Anand, IND; 2 Pharmacology, Pramukhswami Medical College, Karamsad, IND; 3 Biostatistics and Public Health, J. S. Ayurveda Mahavidyalaya, Maganbhai Adenwala Mahagujarat University, Nadiad, IND; 4 Medical Oncology, Manibhai Shivabhai Patel Cancer Centre, Bhaikaka University, Karamsad, IND; 5 Pharmacology, Pramukhswami Medical College, Bhaikaka University, Karamsad, IND

**Keywords:** chemotherapy, depression, geriatric cancer patient, quality of life, social support

## Abstract

Background: Geriatric cancer patients (age ≥65 years) suffer a decline in psychosocial well-being and quality of life (QoL).

Aim: To study social support, depression, QoL domains, and their association with clinicopathological and treatment parameters in Indian geriatric cancer patients.

Methods: QoL, depression, and social support at presentation were studied using the European Organization for Research and Treatment of Cancer Quality of Life Questionnaire Core 30 (EORTC QLQ-C30), Geriatric Depression Scale-4 (GDS-4), and Medical Outcomes Study Social Support Survey (MOS SSS) forms, respectively. An assessment of QoL and treatment outcome against baseline psychosocial domains was done.

Results: A total of 150 patients were enrolled in the study. The mean score for Global Health Status was 53.94; mean scores for Physical, Role, Emotional, Cognitive, and Social Functioning were 77.16, 83.44, 78.39, 86.33, and 69.67, respectively. Global Health Status scores were lower in patients with increasing age, male gender, Eastern Cooperative Oncology Group performance status-2 (ECOG PS-2), BMI ≤18.5, MOS SSS ≤80, renal dysfunction, and head and neck cancer (p-value ≤0.05). A MOS SSS score ≤80 was associated with severe grade Common Terminology Criteria for Adverse Events (CTCAE), recurrence, primary progression, and death (p-value ≤0.05). The mean GDS score was 4.99, with no statistically significant difference in the worst study outcome parameters.

Conclusion: Several clinical and demographic factors significantly influence patient-reported Global Health Status scores. A significant proportion of geriatric cancer patients reported depression and suboptimal social support. Patients with poor social support had a statistically significantly higher incidence of severe grade CTCAE, recurrence, primary progression, and death.

## Introduction

Cancer is a major health concern and a leading cause of morbidity and mortality worldwide. It impacts the physical and psychosocial well-being of patients. Geriatric cancer patients (age ≥65 years) often present with multiple comorbidities, functional limitations, and age-related physiological changes, which influence their treatment tolerance and overall prognosis. Beyond the physical burden of cancer and its associated treatment-related toxicities, geriatric patients frequently experience a decline in health-related quality of life (HR-QoL) and psychosocial well-being [1-4]. These factors can negatively affect treatment adherence, completion rates, and ultimately, survival and disease outcomes [5,6].

HR-QoL encompasses multiple domains, including physical, emotional, cognitive, social, and functional well-being. Geriatric cancer patients frequently experience pain, fatigue, malnutrition, and a decline in physical mobility. Additionally, treatment modalities such as chemotherapy, radiotherapy, and surgical interventions may exacerbate frailty and functional decline, which leads to an increased risk of treatment discontinuation and suboptimal treatment outcomes. The European Organization for Research and Treatment of Cancer (EORTC) Quality of Life Questionnaire Core 30 (QLQ-C30) is a widely used tool/instrument to assess these HR-QoL domains in cancer patients. This QLQ-C30 comprises both multi-item scales and single-item measures. These include five functional scales, three symptom scales, a global health status/QoL scale, and six single items [7-9]. Similarly, depression is a common but often underdiagnosed issue among elderly cancer patients. Studies suggest that depression can influence treatment compliance, worsen symptom burden, and reduce survival rates. Depression in geriatric cancer patients is often multifactorial. Identifying depression early in the cancer treatment process can help healthcare providers implement appropriate psychological and supportive care interventions, improving both mental health and clinical outcomes [10]. Furthermore, depression can lead to a decline in cognitive function and exacerbate physical symptoms, further impairing HR-QoL. The Geriatric Depression Scale (GDS-4) is a validated tool used to screen for depressive symptoms in older adults. Additionally, social support plays a crucial role in determining how well elderly cancer patients cope with their illness and treatment [11-14]. The Medical Outcomes Study Social Support Survey (MOS SSS) is a validated psychometric tool/instrument used to assess four sub-dimensions of social support, including emotional/informational, tangible, affectionate, and social interaction support. It is a 19-item multidimensional, self-administered instrument developed for patients in the MOS [15].

Most research addressing QoL in oncology focuses on younger populations, with geriatric patients often underrepresented in clinical trials [16,17]. There is a pressing need for studies that assess QoL, depression, and social support among geriatric cancer patients undergoing chemotherapy that would guide clinical professionals in decision-making and improve patient care strategies. The present study aims to assess HR-QoL, depression, and social support among geriatric cancer patients undergoing chemotherapy and to analyze their association with demographic, clinicopathological, and treatment-related parameters. The findings of this study would provide valuable insights into the psychosocial challenges faced by elderly cancer patients and highlight potential areas for intervention to improve their overall well-being.

## Materials and methods

This prospective, observational study was conducted at a university cancer hospital and research center in India after obtaining approval from the Institutional Ethics Committee (IEC) in April 2021. This study was part of a PhD study dealing with chemotherapy adverse drug reactions (ADRs) in geriatric cancer patients treated with curative intent. The study aimed to assess HR-QoL, depression, and social support among geriatric cancer patients undergoing chemotherapy and to analyze their association with demographic, clinicopathological, and treatment-related parameters.

Primary objective

To evaluate the baseline prevalence of depression, social support deficits, and HR-QoL impairment in geriatric patients receiving curative-intent chemotherapy.

Secondary objectives

To correlate these baseline psychosocial metrics with the incidence of Common Terminology Criteria for Adverse Events (CTCAE) toxicities, primary disease progression, and overall survival (OS). Patients were enrolled between April 2021 and March 2023 and followed up for a period of one year post-chemotherapy. Written informed consent was obtained from all participants before enrollment. The study adhered to ethical guidelines established by the Declaration of Helsinki, Good Clinical Practice (GCP) guidelines, and the Indian Council of Medical Research (ICMR) regulations.

The study included geriatric cancer patients aged ≥65 years who were undergoing chemotherapy with curative intent. Patients were eligible if they had a histologically confirmed malignancy, an Eastern Cooperative Oncology Group performance status (ECOG PS) of ≤2, and were scheduled to receive chemotherapy as part of their standard treatment. Exclusion criteria included patients receiving palliative chemotherapy, those with cognitive impairment, and those unwilling or unable to provide informed consent.

Patient data were collected systematically using structured case record forms. Baseline demographic information, including age, gender, and socioeconomic status (SES) (assessed using the Modified Kuppuswamy Socioeconomic Scale) [18], was recorded. Clinical examination details such as presenting symptoms, disease duration, type of cancer diagnosis, stage, ECOG PS, nutritional status, and body mass index (BMI) were also documented. Laboratory and radiological investigations were reviewed to establish the clinicopathological profile of each patient. Treatment details, including planned surgery, radiation therapy, and chemotherapy regimens, were noted, along with pre-chemotherapy blood investigations and any modifications in chemotherapy dosing, scheduling, or protocol selection based on initial assessments.

The geriatric assessment, including psychosocial domains and HR-QoL, was done at baseline. HR-QoL was measured using the EORTC QLQ-C30 [9]. Permission to use the EORTC scale was obtained. Mood disturbances, including depressive symptoms, were evaluated using the GDS-4 [13], while social functioning and support were assessed using the MOS SSS [15]. Participants underwent pre- and post-chemotherapy clinical assessments, including investigations as per institutional protocol. Chemotherapy-related ADRs were assessed using the CTCAE v5.0 [19]. GDS-4, Modified Kuppuswamy Socioeconomic Scale, MOS SSS forms, and CTCAE v5.0 are open-access tools available under the public domain. Patients were followed up for one year post-chemotherapy to evaluate survival outcomes. OS was defined as the time from diagnosis to death or last follow-up, while relapse-free survival (RFS) was defined as the time from diagnosis to disease recurrence, primary progression, or death. The impact of baseline QoL, depression, and social support on treatment outcomes was analyzed to identify potential predictors of adverse clinical events.

The sample size was calculated based on two primary outcomes of interest, chemotherapy-related ADRs and mortality. Literature review suggested an estimated chemotherapy-related ADR rate of approximately 30% in older adults globally, while a preassumed ADR rate of 44% was considered for cancer patients in the study setting. To detect this difference with 80% power at a 5% level of significance, a minimum of 99 patients was required. Similarly, for mortality assessment, an estimated global mortality rate of 54% in older adults was compared to a preassumed local mortality rate of 40%, requiring a minimum of 99 patients for statistical significance. Given that the study was conducted during the COVID-19 pandemic, a higher attrition rate (approximately 50%) was anticipated, leading to a planned enrollment of at least 150 patients to ensure sufficient data for analysis. After screening 164 patients, 150 eligible patients were enrolled in the study. At the end of the study in December 2024, OS data for 125 patients were available, with 25 patients lost to follow-up.

Quantitative variables were summarized as mean±SD for normally distributed data and median with interquartile range (IQR) for non-normally distributed data. Qualitative variables were presented as frequencies and percentages. The chi-square test was used to assess associations between categorical variables, while independent sample t-tests and ANOVA were applied to compare quantitative study variables with two or more categories, respectively. MOS SSS scores (questionnaire based on 19 questions of interest), ranging from one to 100, were analyzed both as continuous variables and categorical variables (≤80 and >80), with associations studied using t-tests and chi-square tests. The GDS categories (Normal, Suggestive, and Almost Confirmed) were analyzed for associations with study outcomes using the chi-square test. All statistical analyses were performed using Microsoft Excel (Microsoft Corporation, Redmond, Washington) and IBM SPSS version 25.0 (IBM Corp., Armonk, NY, USA). The p-value of <0.05 was considered statistically significant. Logistic regression was applied to identify factors influencing binary outcome variables, with results presented as odds ratios (OR) with 95% confidence intervals (CI) and corresponding p-values.

## Results

The study cohort predominantly comprised patients aged between 65 and 70 years (64.7%, n=97), with a smaller proportion aged over 80 years (4%, n=6). Males constituted 60% (n=90) of the study population. In terms of SES, the majority belonged to the middle class (54%, n=81), followed by the lower (38.7%, n=58) and upper (7.3%, n=11) classes, as per the Modified Kuppuswamy Socioeconomic Scale. Head and neck cancer (HNC) was the most common malignancy (48.7%, n=73), while gynecological, lung, and genitourinary tract (GUT) cancers were less frequent. Most patients presented with locally advanced disease, with 45.3% (n=68) at stage IVA and 32% (n=48) at stage III. Stage IVa and IVb patients had non-metastatic disease and were treated with curative-intent chemotherapy. The higher percentage of HNC patients represents real-world data from the geographical area where this study was conducted. The study participants were predominantly from lower socioeconomic strata and rural populations and often presented with locally advanced disease due to a lack of awareness of early symptoms and resources. The demographic and clinicopathological characteristics of the study population are summarized in Table 1.

**Table 1:** Demographic and clinicopathological profile *SES as per Modified Kuppuswamy Socioeconomic Scale HNC, head and neck cancer; GIT, gastrointestinal; GUT, genitourinary tract; SES, socioeconomic status

Clinical Characteristics		n (%)
Age	65-70	97 (64.7)
	71-75	39 (26)
	76-80	8 (5.3)
	>80	6 (4)
Gender	Male	90 (60)
	Female	60 (40)
SES*	Upper	11 (7.3)
	Middle	81 (54)
	Lower	58 (38.7)
Diagnosis	HNC	73 (48.7)
	GIT	32 (21.3)
	Breast	22 (14.7)
	Gynecological	14 (9.3)
	Lung	7 (4.7)
	GUT	2 (1.3)
Stage	I	5 (3.3)
	II	22 (14.7)
	III	48 (32)
	IVA	68 (45.3)
	IVB	7 (4.7)

Patients received chemotherapy as per institutional protocol after multidisciplinary team discussion. They received single-agent or combination chemotherapy according to their disease stage indication. Chemotherapy agents included platinum (cisplatin, carboplatin, and oxaliplatin), taxanes (paclitaxel and docetaxel), fluoropyrimidines (5-fluorouracil (5FU) and capecitabine), anthracyclines (doxorubicin), etoposide, gemcitabine, and methotrexate. Patients received supportive care medications as needed, including prophylactic growth factor (granulocyte colony-stimulating factor (G-CSF)) support as per institutional policy. Out of 150 patients, 81 patients (54%) completed all the planned cycles of chemotherapy. In the study, out of 150 patients, a total of nine deaths were reported during and one month post-chemotherapy (CTCAE grade 5). The study population consisted of geriatric patients with comorbidities, and the study was conducted during the COVID-19 pandemic, which could be a confounding factor.

The HR-QoL assessment using the EORTC QLQ-C30 revealed a moderate Global Health Status score with a mean of 53.94 (SD±19.10), indicating a substantial impact of cancer and its treatment on overall well-being. Among the functional scales, cognitive functioning had the highest mean score (86.33), followed by role functioning (83.44) and physical functioning (77.16). Social functioning scored comparatively lower (69.67), reflecting potential challenges in social engagement and support. Notably, the mean score for financial difficulties was 31.56 (SD±22.77), highlighting the financial burden often experienced by geriatric cancer patients.

Additionally, as a component of HR-QoL, we performed symptom scale analysis, which indicated varying degrees of symptom burden among the study participants. The findings revealed that fatigue (31.04), insomnia (31.11), and appetite loss (31.56) were the most frequently reported concerns, indicating a notable impact on patients' daily well-being. Pain was also a significant symptom, with a mean score of 30.11. In contrast, symptoms such as nausea/vomiting, dyspnea, and diarrhea were reported less frequently.

The HR-QoL assessment further revealed that several clinical and demographic factors significantly influenced Global Health Status scores. Notably, patients with advanced age, male gender, ECOG PS 2, lower BMI (≤18.5), renal dysfunction, and HNC had significantly lower Global Health Status scores. These findings highlight the multidimensional challenges faced by geriatric cancer patients, highlighting the need for effective symptom management strategies and the need for tailored interventions to address these vulnerabilities and thereby improve their QoL. The detailed analysis of Global Health Status scores by clinical characteristics is presented in Table 2.

**Table 2:** HR-QoL global health status scores and clinical characteristics *p-value ≤0.05 is statistically significant. SE, standard error; SES, socioeconomic status; ECOG PS, Eastern Cooperative Oncology Group performance status; CCI, Charlson Comorbidity Index; BMI, body mass index; GDS, Geriatric Depression Scale; HNC, head and neck cancer; GIT, gastrointestinal tract; MOS SSS, Medical Outcomes Study Social Support Survey

Parameter	Category	N	Mean	SD	SE mean	95% CI		p-value*
						Lower bound	Upper bound	
Age group	<70	82	80.82	12.08	1.33	78.17	83.47	0.004*
	70-75	54	79.92	11.53	1.57	76.77	83.07	
	>75	14	69.15	13.2	3.53	61.54	76.77	
Gender	Male	90	77.44	12.21	1.29	74.89	80	0.017*
	Female	60	82.35	12.11	1.56	79.22	85.48	
SES	Upper	11	85.45	10	3.01	78.74	92.17	0.243
	Middle	81	78.96	12.11	1.35	76.28	81.64	
	Lower	58	78.89	12.98	1.7	75.47	82.3	
ECOG PS	1	48	83.68	11.27	1.63	80.41	86.96	0.003*
	2	102	77.39	12.39	1.23	74.96	79.83	
CCI	≤2	78	78.57	11.85	1.34	75.89	81.24	0.338
	>2	72	80.32	12.92	1.52	77.28	83.35	
BMI	≤18.50	40	75.07	12.62	2	71.03	79.1	0.009*
	>18.50	110	80.98	11.94	1.14	78.73	83.24	
GDS	≤5	91	79.7	11.67	1.22	77.27	82.13	0.721
	>5	59	78.96	13.46	1.75	75.45	82.46	
Polypharmacy	Yes	48	80.83	12.29	1.77	77.26	84.39	0.336
	No	102	78.74	12.4	1.23	76.3	81.17	
Creatinine clearance	≤60	77	77.32	12.58	1.43	74.46	80.17	0.031*
	>60	72	81.7	11.87	1.4	78.91	84.49	
Location	HNC	73	77.31	12.41	1.45	74.42	80.21	<0.001*
	GIT	32	76.58	12.92	2.28	71.92	81.23	
	Breast	22	90.15	8.43	1.8	86.41	93.89	
	Other	23	79.71	9.31	1.94	75.68	83.73	
MOS SSS	≤80	75	76.92	11.65	1.35	74.24	79.6	0.013*
	>80	75	81.9	12.63	1.46	78.99	84.8	

The assessment of depression was done using the GDS score. The mean (SD) score was 4.99 (2.05), with the majority of participants (n=91, 60.7%) falling within the normal range (score 0-4), while 38.7% (n=58) had scores suggestive of depression (score 5-9), and one patient (0.66%) had a score diagnostic of depression (≥10). Correlation analysis revealed no statistically significant association between GDS scores and key study outcomes, including survival, primary progression, grade 5 toxicity, or maximum CTCAE toxicity grades (Table 3).

**Table 3:** Correlation between GDS scores and clinical parameters GDS, Geriatric Depression Scale; CTCAE, Common Terminology Criteria for Adverse Events

Clinical characteristics		GDS			p-value
		Normal	Suggestive	Almost confirmed (≥10)	
		(0-4 score)	(5-9 score)		
Survival at the end of study (n=125)	Died	36	20	1	0.547
	Alive	38	30	0	
Primary progression (n=150)	No	78	48	1	0.72
	Yes	13	10	0	
Grade 5 toxicity (n=150)	No	85	55	1	0.785
	Yes	5	4	0	
CTCAE grade (n=150)	Mild	61	40	0	0.889
	Severe	31	17	1	

These findings suggest that while symptoms suggestive of depression were present in a notable proportion of patients, they did not significantly influence adverse clinical outcomes in this cohort, which reinforces the need for ongoing mental health assessments in geriatric cancer patients despite the absence of a clear prognostic impact.

The assessment of social support using the MOS SSS revealed a mean overall score of 77.95, indicating a moderate level of perceived social support among the study participants. Domain-specific analysis showed that the mean scores for emotional/informational support, tangible support, affectionate support, and positive social interaction were 34.29, 18.39, 12.64, and 12.63, respectively, reflecting variations in the type and extent of social support experienced. These findings are summarized in Table 4.

**Table 4:** Overall social support and individual social support of different domains MOS SSS, Medical Outcomes Study Social Support Survey

	Overall MOS SSS	Emotional	Tangible support	Affectionate support	Positive social interaction
		Informational support			
Mean	77.9467	34.2933	18.3867	12.64	12.6267
SD	12.382	5.60027	2.15128	2.29759	2.28372

Additionally, we determined the association of social support with clinical outcomes. The detailed correlation analysis is presented in Table 5. Analysis of the MOS SSS scores demonstrated a significant correlation between lower social support scores (≤80) and adverse clinical outcomes.

**Table 5:** Correlation analysis of MOS SSS scores with clinical outcomes *p-value ≤0.05 is statistically significant. MOS SSS, Medical Outcomes Study Social Support Survey; CTCAE, Common Terminology Criteria for Adverse Events

Clinical outcomes		MOS SSS score		P-value*
		≤80.00	>80	
CTCAE_max (n=150)	No	0	2	0.087
	Grade 1	5	14	
	Grade 2	46	34	
	Grade 3	17	21	
	Grade 4	0	2	
	Grade 5	7	2	
Alive (n=125)	Died	32	25	0.030*
	Alive	25	43	
Primary progression (n=150)	No	55	72	<0.001*
	Yes	20	3	
Grade 5 toxicity	No (n=141)	66	75	0.002*

Patients with MOS SSS scores ≤80 had a significantly higher incidence of grade 5 toxicity (P=0.002), primary progression (P<0.001), and mortality (P=0.030). However, no statistically significant association was observed between MOS SSS scores and individual CTCAE grades (P=0.087). These findings suggest the potential impact of reduced social support on disease progression and OS in geriatric cancer patients.

## Discussion

Geriatric cancer patients suffer from geriatric syndrome along with cancer and its treatment-related ADRs. Geriatric syndrome is a complex entity that encompasses clinical conditions such as delirium, falls, frailty, dizziness, syncope, and urinary incontinence that generally do not fit into discrete disease categories. However, its impact on QoL and disability is substantial [20]. A multi-institutional study of HR-QoL and its determinants among cancer patients included 12,148 patients from an Indian database. Older age, lower educational status, chemotherapy, palliative care, surgery, advanced cancer stage, and progressive disease were associated with poor HR-QoL. In this study, the Euro-Qol questionnaire comprising 5 dimensions and 5 levels (EQ-5D-5L) was used to assess QoL. In patients more than 60 years of age, the mean score was 0.524 (95% CI 0.495,0.554) [21]. In our study population, the mean score for Global Health Status was below average (53.94, SD±19.10). The mean scores for Physical Functioning, Role Functioning, Emotional Functioning, Cognitive Functioning, and Social Functioning were 77.16, 83.44, 78.39, 86.33, and 69.67, respectively. There is much attention to the physical domain of QoL, while the other domains, i.e., emotional, spiritual, and social well-being, receive less attention. Social consequences associated with cancer diagnosis and treatment are substantial. There appears to be a great risk of social isolation, in which responses from social relations play an important role. Pooled data from multiple studies showed that up to 45% of cancer patients reported high levels of social difficulty. A lower mean score (69.67) in social functioning was evident in our patient group as well. In our study, Global Health Status scores were significantly lower in patients with increasing age, male gender, ECOG PS 2, BMI ≤18.5, MOS SSS ≤80, renal dysfunction, and HNC diagnosis. Wedding et al. reported that before the start of chemotherapy, depression, functional impairment, and comorbid conditions were associated with poor QoL using the Global Health Status/QoL scale of the EORTC QLQ-C30 [22]. Decoster et al. conducted a multicentric study of prognostic factors for QoL decline in older patients with cancer using EORTC QLQ-C30 in 3,673 patients. In their study, upon multivariate analysis, stage, baseline pain and fatigue, malnutrition, and absence of comorbidities were prognostic for QoL decline [23]. In our patient population, HNC was associated with poorer mean global QoL. In a systematic review of 10 empirical peer-reviewed studies with a primary focus on factors impacting QoL for older HNC patients, Semple et al. reported poorer physical functioning and greater eating and drinking challenges. QoL impacts older patients' decision-making, treatment planning, and intensifies post-treatment support [24].

Earlier studies have looked at the association between depression, all-cause, and disease-specific mortality among older adults. However, studies evaluating a similar relationship in older adults with cancer are very few. Depression is associated with decreased QoL, significant deterioration of recreational and physical activities, relationship difficulties, and sleep disorders. In geriatric cancer patients, it is associated with reduced adherence to anticancer treatment and prolonged length of hospital stay [25]. In our study population, 38.7% (n=58) of patients had scores suggestive of depression (score 5-9), and one patient (0.66%) had a score diagnostic of depression (≥10). In a prospective multicenter cohort composed of incident cases of cancer diagnosed in patients 70 years and older receiving first-line chemotherapy, Duc S et al. studied sociodemographic, clinical, and treatment-related factors of depression. Among 344 patients measured for depression before chemotherapy, 260 had a second assessment at the fourth treatment cycle. At baseline, 45.4% were depressed, and 44.6% were depressed after the fourth cycle. Independent factors of depression were depressive symptoms at baseline (OR=6.7, p<0.001), malnutrition (OR=5.1, p=0.014), and risk of malnutrition (OR=1.6, p=0.014). This study highlighted the role of depressive symptoms and nutritional status at baseline on the occurrence of depressive symptoms during chemotherapy [26].

In our cohort, correlation analysis revealed no statistically significant association between GDS scores and key study outcomes, including disease-free status, survival, primary progression, maximum CTCAE toxicity grades, or death. On the contrary, in a study conducted by Borza et al., depression had a significant impact on mortality. This study was an observational, multicenter, prospective study of 288 patients 70 years or older with cancer followed over 24 months using the GDS-15 scale. After adjusting for cancer-related prognostic factors, a one-point increase in the GDS-15 sum score increased the risk of death by 12%. The authors concluded that more severe depressive symptoms, as measured by the GDS-15, were associated with higher mortality in older patients with cancer [27].

With advancing age, older adults experience a reduction in their social support structure due to life events such as widowhood and retirement. Research suggests that social support has an impact on physical health, well-being, and adjustment to cancer treatment, more so in frail geriatric patients. The MOS SSS is an effective tool for identifying social support needs in older adults with cancer. In our study, the mean MOS SSS was 82.24 (minimum 28, maximum 100, SD±16.26). In a study of 1,460 geriatric cancer patients, Williams et al. reported that 67.5% of patients had at least one unmet social support need. The highest needs were noted in the emotional (49.5%) and physical (47.4%) support subdomains [28].

Social support may also affect the tolerance of older adults with cancer to cancer therapy. In a prospective study of 500 older adults starting a new line of chemotherapy, Dr. Hurria and CARG developed a risk model to predict chemotoxicity. In this model, decreased social activity due to physical health or emotional problems was found to be predictive of an increased risk of chemotherapy toxicity [29]. Similar findings were evident in our study as well. A MOS SSS score of ≤80 was associated with a statistically significantly higher incidence of severe grade CTCAE, recurrence, primary progression, and death.

There are several limitations to our study. It is an observational, single-center study. Patients with metastatic disease were not included. All disease site groups were included in this study, and site-specific subgroup analysis could not be performed. A higher percentage of HNC patients were included in this study, which reflects the higher incidence in the geographical area where the study was conducted. Assessment of QoL, depression, and social support was done only once at baseline. These factors may evolve either way with treatment and time. There were a few potential confounding factors that could not be addressed and may limit the generalizability of the study. Hence, the authors duly recognize that larger, multicentric, interventional, and follow-up studies are the need of the hour to generate further data in this patient population.

## Conclusions

In this observational and single-center study, several clinical and demographic factors significantly influenced patient-reported Global Health Status scores. Fatigue, insomnia, appetite loss, and pain are the most frequently reported concerns, with a negative impact on patients' daily well-being. A significant proportion of geriatric cancer patients reported symptoms suggestive of depression and suboptimal social support. Patients with poor social support had a statistically significantly higher incidence of severe grade CTCAE, recurrence, primary progression, and death. The authors recognize several limitations of this study; however, this study generates important real-world data. Larger, multicentric, interventional, and follow-up studies are the need of the hour to provide optimal care to geriatric cancer patients in the Indian population.
